# Paleogenomics: reconstruction of plant evolutionary trajectories from modern and ancient DNA

**DOI:** 10.1186/s13059-019-1627-1

**Published:** 2019-02-11

**Authors:** Caroline Pont, Stefanie Wagner, Antoine Kremer, Ludovic Orlando, Christophe Plomion, Jerome Salse

**Affiliations:** 1 INRA-UCA UMR 1095 Génétique Diversité et Ecophysiologie des Céréales, 63100 Clermont-Ferrand, France; 2 Laboratoire d’Anthropobiologie Moléculaire et d’Imagerie de Synthèse, CNRS UMR 5288, allées Jules Guesde, Bâtiment A, 31000 Toulouse, France; 3 INRA-Université Bordeaux UMR1202, Biodiversité Gènes et Communautés, 33610 Cestas, France; 4 Centre for GeoGenetics, Natural History Museum of Denmark, Øster Voldgade, 1350K Copenhagen, Denmark

## Abstract

How contemporary plant genomes originated and evolved is a fascinating question. One approach uses reference genomes from extant species to reconstruct the sequence and structure of their common ancestors over deep timescales. A second approach focuses on the direct identification of genomic changes at a shorter timescale by sequencing ancient DNA preserved in subfossil remains. Merged within the nascent field of paleogenomics, these complementary approaches provide insights into the evolutionary forces that shaped the organization and regulation of modern genomes and open novel perspectives in fostering genetic gain in breeding programs and establishing tools to predict future population changes in response to anthropogenic pressure and global warming.

## Introduction

Flowering plants, or angiosperms, have come to dominate terrestrial vegetation. They are an essential component of the carbon, oxygen and water cycles, and paramount to the stability of the climate and substrate of our planet. Through photosynthesis, angiosperms convert solar energy into the basal source of chemical energy that underlies the development of almost all terrestrial ecosystems. Flowering plants are also essential to human society as our principal source of food, animal fodder, medicines, and materials for building, clothing, and manufacturing, among many other uses. Molecular clock estimates [1] and paleontological data [2] suggest that angiosperms emerged some 120–170 million years ago (mya), during a period extending from the Cretaceous to the end of the Jurassic; whereas integrated timescale approaches suggest that they might have emerged even further in the past, some 200–250 mya [3]. Flowering plants rapidly diversified so that over 350,000 species are alive today [4–7]. These species are divided into two main groups, the monocots and eudicots, which account for 20% and 75%, respectively, of the diversity characterized to date [6]. Recent advances in high-throughput DNA sequencing and computational biology have helped researchers to develop the field of paleogenomics, making it possible to retrieve invaluable information about the evolutionary history that underlies the emergence and subsequent diversification of flowering plants.

This research field relies on two main complementary approaches that aim to track the evolutionary genomic changes at both the macro-evolutionary and micro-evolutionary temporal scales. The first, an indirect (or ‘synchronic’) approach, compares modern genomes to reconstruct ancestral genomes over deep timescales of several millions of years (macro-evolution). The second approach, a direct (or ‘allochronic’) strategy, relies on the direct sequencing of genomes from past plant subfossil materials that have been preserved over the past 10,000 years (micro-evolution). Here, we address the underlying methodologies for both paleogenomics approaches, as well as their major achievements and prospects in providing an understanding of the evolutionary trajectories that underpin the genetic makeup of modern plant species.

## Reconstruction of an ancestral genome from modern genome sequences (synchronic reconstruction)

### Background

The recent accumulation of plant genomic resources has provided an unprecedented opportunity to compare modern genomes with each other and to infer their evolutionary history from the reconstructed genomes of their most recent common ancestors (MRCA). Such ancestral genome reconstruction was initially used to investigate 105 million years of eutherian (placental) mammal evolution. The inferred ancestral karyotypes for the eutherians (2n = 44), boreoeutherians (2n = 46), and great apes (2n = 48) were used to increase our understanding of the mechanisms driving speciation and adaptation [8–11]. In particular, eutherian genomes have been found to be surprisingly stable, and affected by only a limited number of large-scale rearrangements during evolution. Higher rates of such chromosomal shuffling have been reported for the branch extending from the great ape ancestor to the ancestor of humans and chimpanzees, which diverged after the Cretaceous–Paleogene (K–Pg) boundary, at a time when the dinosaurs became extinct. Computational reconstructions of mammalian ancestral genomes were instrumental in suggesting that environmental changes may have driven genome plasticity through chromosome rearrangements. These changes may also have led to new variation in gene content and gene expression that gave rise to key adaptive biological functions, such as olfactory receptors [11–13]. Ancestral genome reconstruction has also shed light on plant evolution.

### State-of-the-art methodology

**Fig. 1:**
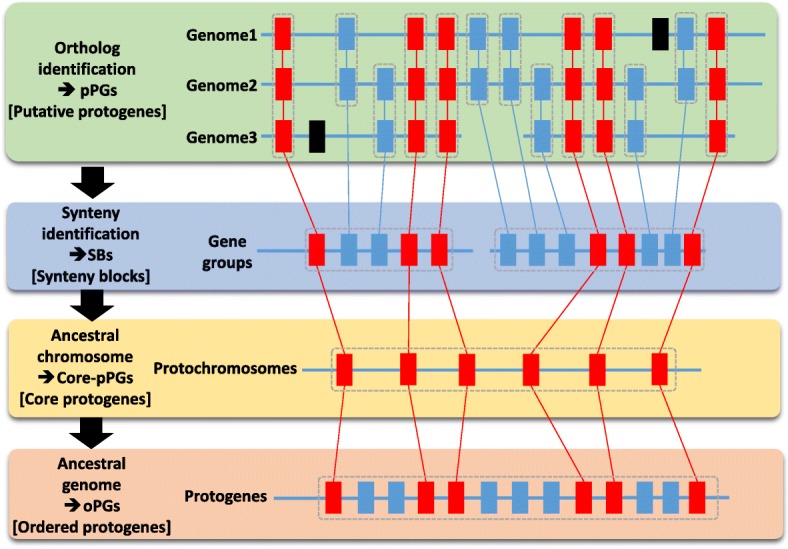
Methodological principles for ancestral genome reconstruction. The four-step strategy is as follows. Step 1: identification of conserved or duplicated genes (putative protogenes (*pPGs*)); here, genes are illustrated as *rectangles* and connected with *red lines* when conserved between all the investigated species or *blue lines* when conserved in a subset of these species. Species-specific genes, which are not present in the inferred ancestor, are shown as *black rectangles*. Step 2: identification of synteny in groups of conserved adjacent genes (synteny blocks (*SB*s)), which are highlighted by *grey dashed rectangles*. Step 3: reconstruction of contiguous ancestral regions (CARs; highlighted by *grey dashed rectangles*) containing genes that are conserved in all of the investigated species (referenced as core-pPGs). Step 4: ancestral genome reconstruction delivering protochromosomes (highlighted by *grey dashed rectangles*) and reordered protogenes (*oPGs*) [23]

The ancestral genome is a ‘median’ or ‘intermediate’ genome consisting of a clean reference gene order that is common to all of the investigated extant species (Fig. 1). The ancestral genomes that are inferred in silico are actually minimal shared ancestral genomes, which lack components of the ‘real’ (unknown) ancestral genomes that were either lost from all of the investigated descendants and/or retained by only one modern species. Such inferred ancestral (minimal) genomes are reconstructed following a four-step strategy [14]. First, sequence comparison across genomes is used to characterize conserved or duplicated gene pairs on the basis of alignment parameters and/or phylogenetic inferences that define genes that are conserved in pairs of species (i.e., putative protogenes (pPGs)). The pPGs that are conserved in all of the investigated species (i.e., core protogenes (core-pPGs)) are used for the definition of synteny blocks (SBs), with the filtering out of groups of fewer than five (pPGs) genes. SBs are then merged on the basis of chromosome-to-chromosome orthologous relationships between the compared genomes, delivering the ancestral protochromosomes (also referred to as contiguous ancestral regions (CARs)). These CARs correspond to independent sets of genomic blocks that display paralogous and/or orthologous relationships in modern species. Finally, the ordering of protogenes (including non-core-pPGs, i.e., genes that are conserved in only a subset of the investigated species) onto the previously defined protochromosomes yields an exhaustive set of ordered protogenes (oPGs).

Putative orthologous (or ancestral) genes that have either been transposed outside of CARs so that they are not conserved in synteny in the course of evolution, or that are only retained in one of the investigated species, or that are lost from all of the investigated species are not identified in SBs and therefore are missing from the inferred ancestral genomes. Several tools such as DRIMM-synteny [15], ADHoRe [16], DiagHunter [17], DAGchainer [18], SyMAP [19], and MCScanX [20] are publicly available for clustering or chaining collinear gene pairs, whereas ANGES [21], MRGA [22], and inferCARs [10] are used for reconstructing ancestral genomes. Finally, the reconstructed ancestral karyotypes can be used to infer a parsimonious evolutionary model that assumes minimal numbers of genomic rearrangements (including inversions, deletions, fusions, fissions, and translocations). Such a model fosters new investigations of the evolutionary fate of ancestral genes/genomes, through precise identification of the changes involved (chromosome fusion, fission, translocation, gains, and losses of genes) and their assignment to specific species or botanical families.

### Major achievements

**Fig. 2:**
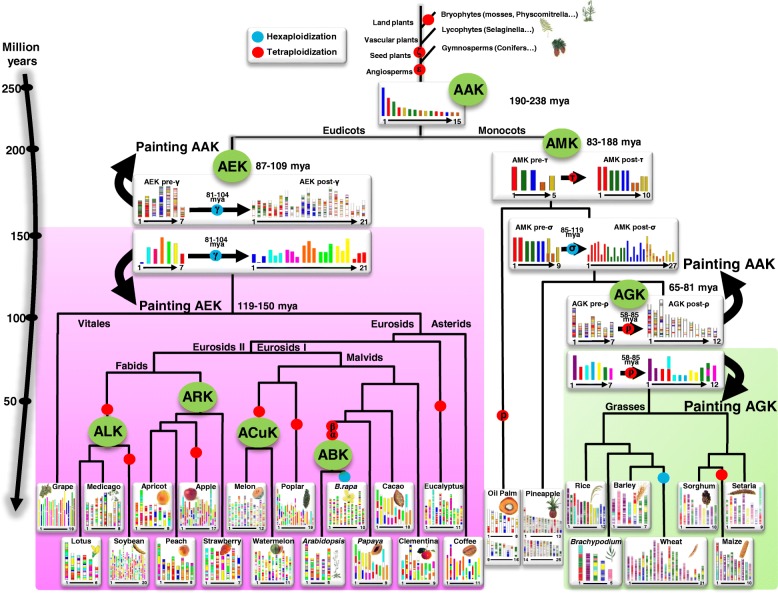
Plant genome evolution from reconstructed ancestors. The present-day monocot (*right side*, with grasses on a *green* background) and eudicot (*left panel*, on a *pink* background) genomes (*bottom*) are represented with color codes to illustrate the evolution of genomic segments from their founder ancestors over the time scale shown on the *left* (in mya). AGK and AEK paintings (*bottom*) represent, respectively, the modern monocot and eudicot genomes based on the ancestral grass karyotype (AGK) and the ancestral eudicot karyotype (AEK) of seven protochromosomes (color code). AAK painting (*top*) represents AGK, AEK, and the ancestral monocot karyotype (AMK), as well as oil palm and pineapple genomes, based on the ancestral angiosperm karyotype (AAK) of 15 CARs (color code). The polyploidization events that have shaped the structure of modern plant genomes during their evolution from inferred ancestors are indicated by *red dots* (duplication) and *blue dots* (triplication). Major angiosperm families are indicated on the tree branches as detailed in Table 1

The ancestral angiosperm karyotype (AAK) has recently been reconstructed with a repertoire of 22,899 ancestral genes that are conserved in present-day crops and that date back 190–238 mya. The angiosperms have also been proposed to emerge some 250 mya using evolutionary timescale approaches [3]. This time period largely overlaps with the late Triassic era and predates the earliest recorded plant fossil [23]. The AAK then diverged, giving rise to the ancestral monocot karyotype (AMK), with five protochromosomes and 6707 ordered protogenes (or seven protochromosomes according to Ming et al. [24]), and the ancestral eudicot karyotype (AEK), with seven protochromosomes and 6284 ordered protogenes [23]. It is possible to reconstruct any investigated modern monocot or eudicot genome using these inferred ancestors (AAK and AMK or AEK), such that modern karyotypes can be seen as a mosaic of reconstructed ancestral protochromosomal segments (Fig. 2). The availability of the AAK, AMK, and AEK helps us to track the evolutionary plasticity acting at the gene, chromosome, genome, and species levels over more than 200 million years of plant evolution [23].

At the gene level, the comparison of the AAK gene repertoire to those of outgroup species, such as gymnosperms, mosses, and single-cell green algae, uncovered genes that are specific to flowering plants. These genes were preferentially assigned to Gene Ontology (GO) terms such as ‘pollen–pistil interaction’, ‘response to endogenous stimuli’, ‘flower development’, and ‘pollination’, corresponding to the key biological processes that drove the transition between gymnosperms and angiosperms [23].

At the genome level, the genomic plasticity inherited through polyploidization events can be assessed, with ~ 60 % of AAK protogenes being present as singletons today in modern species despite recurrent polyploidization events (Fig. 2). This general phenomenon of gene repertoire contraction following polyploidy is also observed at the chromosome level, with a general decrease in chromosome number after whole-genome duplication (WGD) resulting from massive ancestral chromosome fusions through two mechanisms, centromeric chromosome fusion (CCF) and telomeric chromosome fusion (TCF). CCF, which is mainly observed in grasses, involves the insertion of an entire chromosome into a break in the centromeric region of another chromosome. TCF involves the ‘end-to-end’ joining of two chromosomes via their telomeres [25]. The observed general pattern of chromosome number reduction involves unequal reciprocal translocations and the loss of several centromeres, such that only a subset of the ancestral pool of telomeres or centromeres are re-used as functional telomeres or centromeres in modern species [25, 26]. Despite multiple rounds of WGD in the course of plant evolution, the number of genes and chromosomes has been kept constant by massive diploidization and fusion events, at the gene and genome levels, respectively. Diploidization did not occur at random in the genome, particularly where retained ancestral genes were partitioned between paralogous blocks so as to form ‘most fractionated’ (MF, also known as S for sensitive) and ‘least fractionated’ (LF, also known as D for dominant) chromosomal compartments [14]. ‘RNA binding’, ‘nucleic acid binding’, ‘receptor activity’, ‘signal transducer activity’, ‘receptor binding’, and ‘transcription factor activity’ are frequent GO terms associated with molecular functions that are enriched in extant genomes relative to the AAK. They correspond to adaptive or specialized biological functions for which multiple copies of genes were conserved after WGD and have survived the general diploidization phenomenon [23].

As has been proposed for mammalian evolution, paleopolyploidy events in angiosperms are usually considered rare, are likely to lead to an evolutionary dead-end, and may have served as the basis for species diversification and survival during episodes of mass species extinction [27–29]. Although still debated, the ancient paleopolyploidization as well as ancestral speciation events in angiosperms may have been associated with known periods of species extinction, such as the Cretaceous/Paleogene (called K-Pg, ~ 65 mya) transition [27] or the Triassic/Jurassic (called Tr-J, ~ 200 mya) transition [30]. More recent paleopolyploidization events that are specific to plant lineages (or even species) may be associated with more recent plant diversification periods during the Paleogene and Neogene (~ 20–30 mya), as observed from historical changes in dry forest communities and biomasses [31, 32]. Thus, polyploidy appears to have played a major role in (re-)shaping structural and functional genomic diversification during angiosperm evolution, with contrasting rates of changes between species, subgenomes, genes, and functions. It may also have delivered biological novelties that have enhanced tolerance of environmental changes, including those occurring during mass extinction events.

### Grasses as a case study

Besides the recovery of extinct AMK, AEK, and AAK founder karyotypes, the synchronic approach has also enabled the computational reconstruction of the ancestral genomes of major angiosperm lineages. In eudicots, ancestral genomes have been proposed for the Rosaceae [33], Brassicaceae [34], and Cucurbitaceae [35] subfamilies, consisting of nine, eight (or seven), 12 (using the melon genome as pivot) protochromosomes, respectively, as well as for the legumes [36]. In grasses, the ancestral grass karyotype (AGK), which takes into account gene conservation between rice, wheat, barley, *Brachypodium*, sorghum, setaria, and maize, was structured into seven protochromosomes containing 8581 protogenes (9430 in Wang et al. [37]) and with a minimal gene space physical size of 30 Mb [23, 38, 39]. This ancestral genome went through a paleotetraploidization event (involving seven duplicated blocks shared by modern monocots) more than ~ 95 mya [37, 38, 40]. Two subsequent symmetric reciprocal translocations, one of which was centromeric (CCF) and the other telomeric (TCF), and two asymmetric reciprocal translocations resulted in a total of 12 chromosomes [23, 39] bearing 16,464 protogenes (18,860 according to Wang et al. [37]). All investigated modern grass genomes can then be reconstructed from this post-polyploidy ancestral karyotype of 12 protochromosomes, taking into account CCF, TCF, translocation, and inversion events (Fig. 2). Rice has retained the n = 12 structure of the AGK and has been proposed to be the slowest evolving species among the grasses [23, 37], whereas the other species underwent numerous chromosome rearrangements to reach their present-day karyotypes [23, 38, 39]. Rice can, therefore, be considered as a reference genome (also known as a ‘pivot’) for comparative genomics studies in grasses.

The grasses appear to constitute a key botanical family in which to investigate the role of polyploidizations in promoting species speciation and adaptation. Grasses experienced an ancestral paleotetraploidization event as well as species-specific polyploidization events, with a tetraploidization event in maize and tetraploidization or hexaploidization events in wheat. After a polyploidization event, homoeologous chromosome differentiation is necessary to stabilize meiosis by preventing incorrect pairing between homoeologs. This is achieved through massive partitioning of the organization and regulation of the subgenomes, involving the fusion, fission, inversion, and translocation of chromosomes, loss of genes or DNA, and neo- or sub-functionalization of gene pairs. Ultimately, such post-polyploidy genomic plasticity led to novel phenotypes that underlie the evolutionary success of polyploid plants and, ultimately, was selected for by humans during domestication (reviewed in [14, 29, 41, 42]).

### Promising scientific avenues from inferred ancestral genomes

Inferred ancestral genomes are not only crucial for understanding how plant genomes have evolved at the chromosome and gene scales, but also offer the possibility to address, in novel ways, issues regarding translational research and post-polyploidy plasticity that are relevant to plant breeding.

#### Translational research

Ancestral genomes and related comparative genomics data are delivered through public web servers such as PlantSyntenyViewer (https://urgi.versailles.inra.fr/synteny [30, 34, 39]), Genomicus (http://www.genomicus.biologie.ens.fr/genomicus-plants [43]), COGE (https://genomevolution.org/coge/ [44]) and PLAZA (http://bioinformatics.psb.ugent.be/plaza/ [45]). The ancestral genomes (AAK, AEK, AMK, and AGK, as well as ancestral genomes for the Rosaceae, Brassicaceae, and Cucurbitaceae; Table 1) provide a list of accurate orthologs between species that can be used to improve the structural and functional annotation of genomes. The plant genomes shown in Fig. 2 have been sequenced, assembled, and finally annotated by different methods and groups, potentially resulting in some inconsistencies. With the use of reconstructed ancestral genomes, structural (intron and exon structure) and functional (GO) annotations of genes can be improved by comparing orthologous and paralogous gene sets that may share similar (ancestral) genomic features. Reconstructed ancestors can also be used as a useful resource for translational research on key agronomical traits, particularly from model species (such as *Arabidopsis thaliana*) to crops [46]. Modern monocot and eudicot crops can now be connected via the 22,899 protogenes that define the AAK [23], offering the opportunity to exploit the knowledge gained on genes underlying traits of interest in models based on orthologs or paralogs in crops delivered in the proposed evolutionary scenario and associated paleogenomic data (Fig. 2). Such translational-based dissection of traits has been performed successfully in several botanical families, including legumes (for example, between *Medicato truncatula* and pea, as described by Bordat et al. [47]) and grasses (for example, between *Brachypodium distachyon* and wheat, as described by Dobrovolskaya et al. [48]).

**Table 1:** Ancestral plant genomes

Family	Dating	Name	Chromosome number	Gene number	Reference
Angiosperms	190–238	AAK (post-ε/ζ)	15	22,899	[23]
Eudicots	87–109	AEK (pre-γ)	7	6284	[23]
Eudicots	87–109	AEK (post-γ)	21	9022	[23]
Monocots	100–150	AMK (pre-τ)	5	6707	[23]
Monocots	100–150	AMK (post-τ)	10	13,916	[23]
Grasses	65–81	AGK (pre-ρ)	7	8581	[39]
Grasses	70–96	AGK (pre-ρ)	7	9430	[37]
Grasses	65–81	AGK (post-ρ)	12	16,464	[39]
Grasses	70–96	AGK (post-ρ)	12	18,860	[37]
Brassicaceae	27–40	ABK (post-α/β)	8	20,037	[34]
Brassicaceae	23–27	ACaK (post-α/β)	8	22,085	[34]
Brassicaceae	23–27	PCK (post-α/β)	7	21,227	[34]
Rosaceae	70–90	ARK (post-WGD)	9	8861	[33]
Cucurbitaceae	25–50	ACuK (post-WGD)	12 *(Melon as pivot)*	18,534	[35]
Legumes	56–59	ALK (post-WGD)	–	28,900	[36]

Summary of reconstructed ancestral angiosperm genomes listing the targeted botanical family, dating (in mya) of the whole-genome duplication defining the delivered post- and pre-polyploidization ancestors, ancestral genome name, number of chromosomes, number of genes and associated references in the literature

*Abbreviations*: *AAK* ancestral angiosperm karyotype, *ABK* ancestral *Brassicaceae* karyotype, *ACaK* ancestral *Camelineae* karyotype, *ACuK* ancestral *Cucurbitaceae* karyotype, *AEK* ancestral eudicot karyotype, *AGK* ancestral grass karyotype, *ALK* ancestral legume karyotype, *AMK* ancestral monocot karyotype, *ARK* ancestral *Rosaceae* karyotype, *PCK* proto-*Calepineae* karyotype, *WGD* whole-genome duplication

#### Polyploidization

Polyploidization events have been proposed as a major source of genetic novelty during evolution. Such post-polyploidy genomic plasticity takes place in paleopolyploids that are subject to diploidization (evolution toward a reduction of duplicate redundancy) through (not exclusively): (i) differences in ancestral gene retention yielding contrasted plasticity between MF (or S) and LF (or D) compartments; (ii) bias in GO for the retention of multiple copies of genes displaying an enrichment in functional categories such as transcriptional regulation, ribosomes, response to abiotic or biotic stimuli, response to hormonal stimuli, cell organization, and transporter functions; (iii) partitioned gene expression with differences in transcript abundance or neo- and sub- functionalization patterns between retained pairs; (iv) contrasted single nucleotide polymorphisms (SNPs) at the population level between paralogous genomic fragments; and (v) contrast in small regulation as well as differences in epigenetic (CG methylation) marks between duplicated blocks/genes [49]. Such subgenome dominance phenomena, which partition the organization and regulation of diploidized paleopolyploids, have been particularly exemplified in Brassicaceae and maize [50–53] but are reportedly so far undetectable in soybean, banana, and poplar [54].

The evolutionary plasticity gained from recurrent polyploidization and diploidization (also known as post-polyploidization diploidization (PPD) [55]) processes has provided the basis for functional and phenotypic novelty in angiosperms. This plasticity may underlie a plant's ability to survive in or invade a novel environment, ultimately driving the observed evolutionary success of important plant families [27]. Nevertheless, the continuum and interplay between the reported structural and functional reprogramming after PPD processes remain poorly understood. The access to ancient DNA (aDNA) sequences from extinct diploid and polyploid ancestors contemporary to past polyploidization events will further expand our understanding of this major phenomenon driving plant evolutionary dynamics, making it possible to characterize the driving molecular mechanisms that have potential for use in breeding. In that regard, nascent polyploids (particularly in wheat and Brassicaceae) provide opportunities for testing the hypothesis that polyploidization accelerates evolutionary adaptation to environmental changes [56].

## Evolutionary processes inferred from ancient DNA (allochronic reconstruction)

### Background

Ancient DNA sequencing offers a unique opportunity to retrieve genetic information from past individuals. It has been applied successfully to ancient hominins and human individuals (see review in Marciniak and Perry [57]), and to a handful of mammal species, including woolly mammoths [58], aurochs [59], horses [60], and dogs [61], at both the genomic and the population scale. Ancient genomes have helped to unveil the complex population dynamics and processes that underlie evolution, involving admixture, migration, and adaptation [62–64]. Signatures of adaptation in response to natural or human-driven selection are embedded within the genomes of modern populations and species [65], making inferences about past selective processes possible. Such indirect approaches have clearly demonstrated the power of natural and artificial selection in shaping local adaptation, but have also shown limitations because evolutionary inferences are based on theoretical models with simplifying assumptions. The possibility of adding a temporal dimension to such analyses, overlapping key evolutionary transitions such as demographic or environmental changes, could provide enhanced statistical power for detecting and quantifying the genomic changes underlying adaptive [66, 67] and non-adaptive histories [68, 69]. Such studies are still embryonic in plants but will eventually help us to (i) chart the complex patterns of adaptation through space and time, and (ii) measure the evolutionary responses of plants to key evolutionary and/or environmental transitions.

### State-of-the-art methodology

**Fig. 3:**
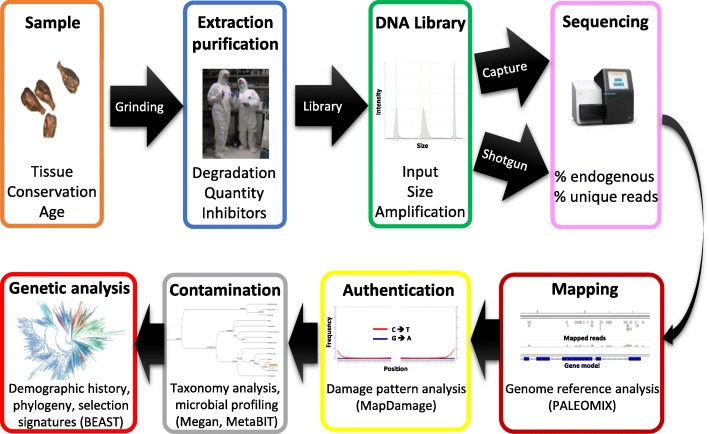
Methodological principles for ancient DNA investigation. Documentation of tissue type (i.e., leaves, seeds, stems, wood), conservation (i.e., dessicated, waterlogged, charred), and age is followed by sample decontamination, DNA extraction (depending on DNA degradation, DNA size, quantity, and absence of inhibitors), and NGS library (single-stranded) preparation (depending on input quantity, aDNA fragment size, and the number of amplification cycles) performed in standardized clean conditions using laboratory procedures optimized for aDNA. Sequencing reads can be mapped against a reference genome (defining endogenous aDNA and unique mapped reads) and authenticated based on typical post-mortem damage patterns using bioinformatic pipelines tailored for aDNA. The exogenous DNA content can be evaluated using metagenomic analysis tools. Finally, the authenticated endogenous DNA can be compared to modern reference samples to unveil genomic footprints of species’ origin, migration, anthropogenic translocation, extinction, and hybridization events. Examples of bioinformatic tools used in investigating aDNA sequences are mentioned at the bottom of each panel (see main text for further details)

In addition to working under rigorous clean laboratory conditions, appropriate sequencing and computational methods are required to authenticate and analyze aDNA sequences correctly (Fig. 3). Over the past decade, aDNA research has moved from the characterization of short pieces of DNA, mostly mitochondrial, to complete genome sequencing (for a review, see Orlando et al. [70]). This impressive progress has been made possible due to the advent of high-throughput DNA sequencing platforms, which can sequence up to several billions of short nucleotide reads in no more than a few days [71, 72]. Such sequencing capacities have opened access to even the most minute fraction of DNA molecules preserved in fossil specimens, even though these molecules are often outnumbered by DNA fragments from environmental microbes [73].

Current aDNA methodologies do not rely on brute-force sequencing of DNA extracts but rather leverage the specific biochemical features of aDNA molecules in subfossils. First, a number of paleontological and archaeological remains, such as hair [74], petrous bones [75], and tooth cementum [76], generally provide micro-environments with DNA preservation conditions that are generally better than those provided by other types of calcified remains, such as shells [77]. Second, DNA extraction methods tailored to the retrieval of the most fragmented DNA templates, which also represent the most abundant fraction of ancient DNA molecules, have been developed [78, 79]. As the information present in 25–35 bp fragments is generally compatible with accurate sequence alignment, the recovery of such ultra-short templates has greatly improved the sensitivity of aDNA analyses. Third, some extraction procedures, including pre-digestion [80], the washing steps prior to full digestion [81], and other techniques [82, 83], have proved useful for removing at least a fraction of environmental contamination.

In addition, a range of DNA library construction methods have also been developed, and important biases have been mitigated, including those that occur during adapter ligation [84] and PCR amplification [85]. The development of DNA library construction methods that exploit molecular features of aDNA, in particular the presence of damage in the form of single-strand breaks [86] and/or deaminated cytosines [87], has also enhanced our ability to access aDNA templates. Finally, target enrichment approaches, aimed at the characterization of organellar DNA [88] or of a limited number of mitochondrial and nuclear loci [89], or up to hundreds of millions of SNPs scattered throughout the nuclear genome [90] and even of the entire nuclear genome [91, 92], now contribute to the retrieval of the genome-scale information required to address major biological questions in both a cost- and time-effective manner. It is worth noting that a number of computational approaches have also helped to quantify DNA damage [93, 94], to reduce its impact on downstream analyses [94, 95], and to improve the sensitivity and accuracy of aDNA read alignments [96–99]. Unlike material from human or vertebrate taxa, plant material contains polyphenols, polysaccharides, and other molecules that can interfere with standard molecular tools and/or reagents. Therefore, the development of procedures that are tailor-made for the recovery, purification, and manipulation of DNA from botanical remains is necessary (Table 2).

**Table 2:** Ancient plant DNA

Species	Dating^a^	Site	Sample	Characterization^b^	Extraction^c^	Reference
Oak	500–9800 BP	Europe	Waterlogged wood	NGS	TrisHCL SDS CaCl_2_ EDTA DTT PK/Phchlo/Column	[142]
Japanese cedar	3600 BP	Japan	Buried tree	PCR/sequencing	Column	[133]
Maize	1100–6000 BP	New World	Desiccated cob	PCR/sequencing	CTAB TrisHCL NaCl EDTA/Chlo	[111] [112]
Maize	360–1320 BP	New World	Desiccated cob	PCR/sequencing	Column	[114]
Maize	670–5280 BP	New World	Desiccated cob	Capture, NGS	SDS DTT PK/Phchlo/Column	[115]
Maize	650–4300 BP	New World	–	PCR/sequencing	SDS DTT PK/Phchlo/EDTA PTB/Column	[113]
Maize	4700 BP	Chile, Peru	Charred and non-charred grain	PCR/sequencing	SDS DTT PK/Phchlo	[110]
Maize	5310 BP	Mexico	Desiccated grain	NGS	TrisHCL SDS CaCl_2_ EDTA DTT PK/Phchlo/Column	[116]
Maize	5300 BP	Mexico	Desiccated cob	NGS	–	[117]
Sunflower	3100 BP	USA	Desiccated disk fragment, pericarp, kernel	NGS	TrisHCl NaCl SDS CaCl_2_ EDTA DTT PK/Phchlo/Column	[119]
Radish	350–550 AD	Egypt	Desiccated seed	Chemical analysis/PCR/sequencing	CTAB TrisHCL NaCl EDTA/Chlo	[124]
Sorghum	2800 BP	Egypt	Desiccated seed	PCR/sequencing	–	[125]
Rice	1200–2400 BC	China	Desiccated seed and chaff	PCR/sequencing	Magnetic beads	[127]
Grape	1600–2500 BP	Europe	Waterlogged and charred pip	PCR/sequencing	DTAB /Chlo/CTAB	[120]
Grape	7^th^–15^th^ century AD	Italy	Waterlogged pip	PCR	SDS DTT PK/Phchlo/Column	[121]
Grape, maize, olive, dogwood, cotton	400–2400 BP	New World, Europe	Non-carbonized remain	PCR/sequencing	SDS DTT PK/Phchl	[122]
Barley	6200–5800 BP	Israel	Desiccated seed	NGS	CTAB TrisHCL PVP βME/Phchlo/Column	[104]
Barley	3000 BP	Egypt	Desiccated grain	PCR/sequencing	SDS DTT PK EDTA PTB/Column	[103]
Barley, wheat	150–5250 BC	Spain	Charred, partially charred, waterlogged seed	PCR/sequencing	TrisHCL SDS EDTA PK/Phchlo or Column	[105]
Wheat	700 AD–8400 BP	Anatolia	Charred grain	PCR/sequencing	CTAB TrisHCL NaCl EDTA/Chlo	[109]
Wheat	340–3500 BP	Spain	Charred and desiccated seed	PCR/sequencing	Tris EDTA CTAB βME/Column	[108]
Cotton	750–3750 BP	Brazil, Peru, Egypt	Desiccated seed	NGS	CTAB/Column	[132]
*Arabidopsis*	300 BP	USA	Herbarium	NGS	CTAB or PTB DTT/Column	[131]

Summary of ancient plant nuclear DNA recovery listing species, dating, location site, sample type, characterization method, extraction protocol, and associated references. ^a^Datings are referenced as in the publication concerned using BC, AD, or BP. ^b^*PCR/sequencing* polymerase chain reaction and sequence capture, *NGS* next-generation sequencing. ^c^*DDT* dithiothreitol proteinase K, *Phchlo* phenol-chloroform, *Ph* phenol, *Chlo* chloroform, *SDS* sodium dodecyl sulphate, *EDTA* ethylene-diamine-tetraacetic acid, *CTAB* cetyltrimethylammonium-bromide, *PTB* phenacylthiazolium bromide*, DTAB* dodecyltrimethylammonium bromide, *βME* β-mercaptoethanol, *Tris* tris(hydroxymethyl) aminomethane, *PK* proteinase K

### Major achievements

Plant seeds can provide a favorable environment for the preservation of nucleic acids over millennia, perhaps as a result of the active desiccation mechanisms involved in dormancy [100, 101]. While recent work has shown encouraging results, studies leveraging the information in the DNA (and/or RNA) fragments present in plant subfossils are still scarce (for review, see Gutaker and Burbano [102]). The number of the species studied spans a large taxonomic range and includes barley [103, 104], wheat [105–109], maize [110–118], sunflower [119], grape [120–122], bottle gourd [123], radish [124], sorghum [125], papyri [126], rice [127], olive [128], orchid [129], *Prunus* [130], *Arabidopsis* [131], cotton [132], and trees [133–136]. Similarly, the primary material used for DNA extraction includes a whole variety of tissues, such as fruits, seeds, leaves, and woods, preserved in a wide range of conditions, including charred, waterlogged, desiccated, or mineralized remains. Ancient DNA from organelles, which have sequences that are highly conserved among plant species and which is generally better preserved than the nuclear genome, have been widely used in paleogenomics studies on plants over the past decade [137]. Such organellar DNAs include ribosomal (rDNA) and chloroplast (cpDNA) markers such as the *rbcL* gene (which encodes the large subunit of ribulose-1,5-bisphosphate carboxylase, an important enzyme in photosynthesis), trn introns and spacers (which offer more variable non-coding information), and *matK* (the maturase K) gene. The internal transcribed spacer 1 of the ribosomal DNA gene (*ITS1*) has classically been used in characterizing plant aDNA in papyri [126], *Prunus* [130], bottle gourd [123], orchid [129], olive [128], wheat [105, 106], and trees [134–136]. Importantly, in contrast to studies on animals, mitochondrial DNA (mtDNA) has been overlooked in plant aDNA research, probably because of its more-than-100-times-slower mutation rate [138–140].

At the nuclear level, a handful of genetic markers, mostly carrying functional variants that are associated with flowering time and starch storage, have already been characterized, originally by PCR, and more recently by target-enrichment approaches, which helped track the genetic variation of major maize genes over the past 6000 years [116]. The presence of lipids (fatty acids and sterols) and nucleic acids in desiccated radish seeds from a 6^th^ century storage vessel recovered from Qasr Ibrîm in Egypt has been reported [124]. Further DNA investigation of plant remains from archaeological sites in Egypt have been also reported for sorghum [125]. In rice, genomic sequences from remains found at Tianluoshan, a site of the local Hemudu Neolithic culture in the low Yangtze, were compared to current domesticated and wild rice populations in order to investigate the genetic changes underlying the domestication syndrome [127]. Microsatellite loci have also been used to investigate the origins of grape seeds preserved by waterlogging and charring at several European Celtic, Greek, and Roman sites [120, 122]. Cotton aDNA was used to investigate changes in transposon composition that have occurred over the past 1600 years of domestication [132]. Finally, moving towards more recent times, herbarium specimens have been found to generally yield excellent DNA preservation, compatible with the whole-genome sequencing of plants such as potato [141], *Arabidopsis* [131], or orchid [129], and even of some of the pathogens responsible for historical famines such as that caused by potato blight [141]. More recently, technological advances in next-generation sequencing (NGS; Table 2) as well as target enrichment approaches (such as sequence capture) have delivered highly relevant retrieval and authentication of aDNA in barley [104], maize [115–117], oak [142], and sunflower [119], which can now be used as standards for plant aDNA studies. In addition, the recent recovery of aDNA from waterlogged wood (oak) remains has opened up new avenues for investigating the recent evolution of forest cover in the face of climate and/or anthropogenic changes [142].

The most thorough plant aDNA studies have been probably carried out in maize and barley. In maize, the analysis of transposable elements (*Mu*) in pre-Columbian kernels [111], and of the alcohol dehydrogenase gene (*adh*) and microsatellite loci in desiccated maize cobs excavated in caves, provided the first insights into maize origin and domestication [112, 114]. Then, target enrichment through gene capture helped to decipher the early diffusion of maize into the American Southwest and to track genomic selection signals during different phases of domestication, in particular in loci relevant to drought tolerance and sugar content [115]. In barley, the genome (exome) sequence of 6000-year-old barley grains from (pre-)historic caves in the Judean Desert revealed close affinities with extant landraces from the Southern Levant and Egypt. These findings were consistent with a proposed origin of domesticated barley in the Upper Jordan Valley, as well as with gene flow between cultivated and wild populations during the early domestication phase [104]. In addition to aDNA, plant subfossils can also provide ancient RNA (including small RNAs) and epigenetic (i.e., DNA methylation) signatures [143–146]. Barley is the ancient crop that has garnered most of the attention at the DNA, RNA, and epigenetic levels. A series of investigations have addressed the complex process of local adaptation and domestication, or have identified the presence of barley stripe mosaic virus, one of the major diseases affecting this major crop [101, 144–146].

### Wheat as a case study

**Fig. 4:**
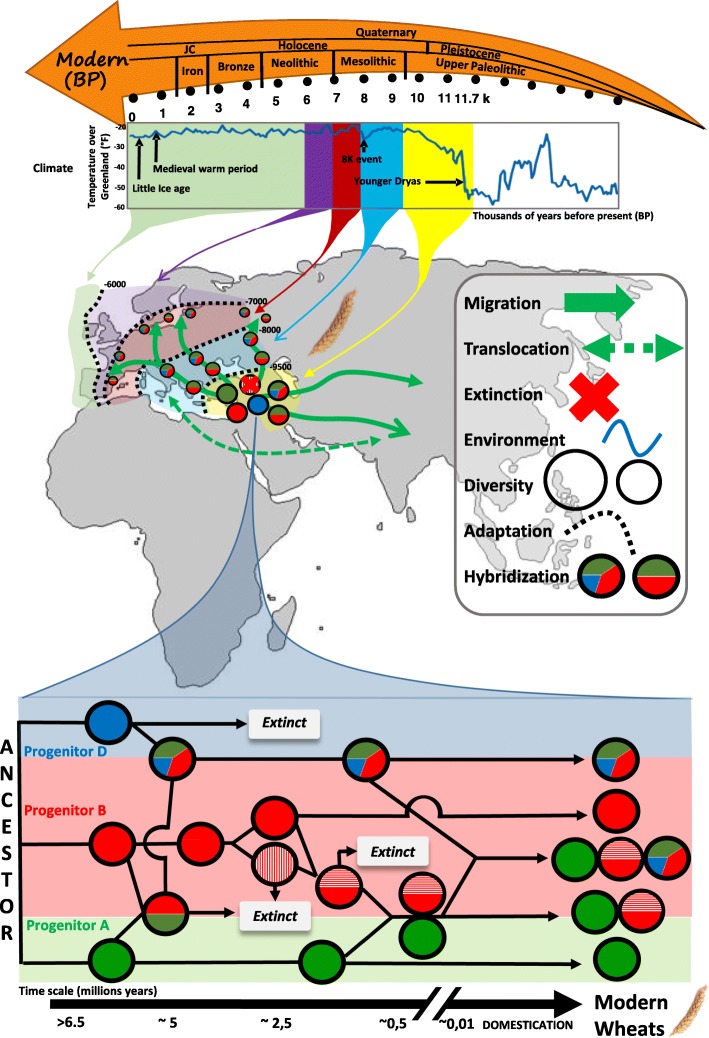
Scientific insights gained from plant aDNA, as exemplified by wheat. *Top*: The geological periods are indicated at the top (*orange arrow*) above the associated temperature profiles (*blue curve* showing the variation of temperature at constant elevation (y-axis) over thousands of years before present (x-axis), modified from Cuffey and Clow [168]) and key climatic changes. *Center*: The known historical routes of wheat migration from the site of origin (Fertile Crescent) are indicated by *green arrows*: westwards via an inland route (through the Balkans to Central Europe) or along a coastal path (via Anatolia to the Mahgreb and Iberian peninsula); or eastwards via routes to the north and along the Inner Asian Mountain Corridor. Major migration phases are shown in different colors on the time scale. Diploid, tetraploid, and hexaploid wheats are depicted as *colored circles* (*green*, *red*, and *blue colors*) with mixed colors reflecting hybridization events. *Bottom*: Illustration of the hexaploid bread wheat paleohistory from progenitors (with some possibly extinct) A (*green circles*), B (*red circles*), and D (*blue circles*) shown at the *left*, along the time scale expressed in million years (*left*) at the bottom. Subgenomes (A, B, and D) are illustrated with *circles* so that hybridization events are highlighted with mixed color within *circles* (similar to the central panel). Modern (diploid, tetraploid, and hexaploid) species are illustrated at the *right* of the figure. Wheat aDNA offers the opportunity to investigate the impact of migration, translocation, extinction, and hybridization events in shaping the modern genetic diversity and in driving adaptation to environmental constraints (temperature variation) over 10,000 years of domestication and cultivation

Plant aDNA provides a catalog of ancient sequence polymorphisms that are extremely valuable in disentangling the temporal and geographical locus of domestication as well as the patterns of migration (diffusion). This catalog also helps to detect hybridization events between cultivated and wild relatives, including those that may have been advantageous. More than 2500 plant species are thought to have been domesticated over the past 12,000 years of evolution, since the last glacial period [147]. However, the domestication history of most crops is still contentious and several evolutionary models have been proposed. Ancient DNA has provided the data necessary to test a number of competing scenarios, mostly in cereals and especially in wheat, pertaining to the migration, translocation, extinction, hybridization, and demographic dynamics underpinning modern cultivars (Fig. 4).

It is currently thought that tetraploid wheats, which are used for pasta production, emerged some 0.5 mya from the hybridization of a wild *Triticum urartu* Tumanian ex Gandivan (AA) and an undiscovered species of the *Aegilops speltoides* Tausch lineage (BB). It is widely accepted that the domestication of this tetraploid wheat (wild emmer AABB) in southeastern Turkey—within the so-called ‘Fertile Crescent’ (a region extending from western Iran, Iraq, Jordan, Israel, Lebanon, and Syria to south-east Turkey)—followed by a north-eastern migration, led to its hybridization with *A. tauschii* (DD) and the emergence of bread wheat (*Triticum aestivum*, AABBDD). This hybridization event most probably occurred within a corridor spanning from Armenia to the south-western coast of the Caspian Sea [148, 149]. After the last glacial maximum, human sedentarism associated with the development of agriculture emerged in several sub-regions of the Fertile Crescent [150], where several major crops, including wheat and barley, and farm animals such as sheep, goats, cows, and pigs were domesticated.

Plant fossil remains displaying the characteristics of domesticated cereal crops have been discovered at multiple archaeological sites dating from 8000–10,000 years ago, a key historic period marking the human transition from a foraging lifestyle to early sedentary agricultural societies. The domestication of wheat involved selection for traits that are related to seed dormancy and dispersal, such as brittle rachis, tenacious glume, and non-free-threshing traits [151]. The spread outside of the original domestication center followed four major historical routes of human migration, including a westwards expansion through inland (via Anatolia and the Balkans to Central Europe) and coastal (via Egypt to the Mahgreb and Iberian peninsula) paths, and an eastwards expansion through the north and along the Inner Asian Mountain Corridor [152]. Following domestication, modern breeding activities starting after 1850 CE (Common Era) further reduced the genetic diversity in genomic regions harboring genes involved in agricultural performance or adaptation (such as photoperiodism, vernalization, flowering, accumulation of seed storage protein, plant architecture, and so on) [153].

Access to aDNA would enable the estimation of the loss of genetic diversity associated with the various processes operating during 10,000 years of domestication, hybridization, migration, and adaptation. Among the evolutionary processes underpinning the origins of wheat, hybridization (often referred to as reticulated evolution) remains controversial because of the lack of a modern representative of some diploid progenitors (especially for the B subgenome; Fig. 4). Future aDNA work, notably for the so-called naked wheat that was common in the Neolithic [154–156] or the ‘new glume wheat’ found at Neolithic and Bronze Age sites [157] and proposed to be an extinct wheat cultivar [158], may shed light on such controversies. Wheat aDNA recovered from waterlogged, desiccated, and (semi-)charred remains (grains, rachides, and/or spikes), provides a seminal resource for attempts to address wheat origin and evolution during the past 10,000 years of domestication [106–109, 159], as well as the impact of polyloidization (comparing diploid, tetraploid, and hexaploid wheats) in adaptation. Nevertheless, the extraction of aDNA from wheat remains is still challenging because of the diversity of the tissues considered and their conservation over time. The two different methods, classically used for plant aDNA extraction (proteinase K or cetyltrimethylammonium-bromide (CTAB); Table 2), still need to be refined for the retrieval of DNA from all types of plant remains, especially charred grains. Although charred seeds retain their morphological characteristics and are therefore suitable for botanical classification, the preservation of DNA within such materials remains so far unlikely [160].

### Promising scientific avenues from sequenced ancient DNA

Beside solving phylogenetic questions regarding evolution, admixture, hybridization events, relationships between species or populations, domestication, and improvement processes, aDNA can provide new possibilities for addressing a number of issues related to plant adaptation and diversification.

#### Adaptation

Ongoing climate change, the steady growth of the human population worldwide, and increasing demand from emerging economies place food security at threat all over the world [161]. In addition, the demand for wood is growing continuously at a time when forest trees are exposed to rapidly increasing biotic and abiotic threats [162, 163]. These issues are of utmost importance in the context of ongoing climatic changes [164] and the rise in food and wood demand from the expanding world population [165]. The development of high-yielding, durably stress-resistant crops and trees is thus paramount for the sustenance of future human societies. This challenge can be addressed in part through the identification, conservation, and exploitation, through genome-informed conservation and breeding strategies, of key genetic polymorphisms that enhance plant resilience in the face of environmental pressures [166]. Plants have faced temperature and water constraints in the past, including some similar to the +0.3°C to +4.8°C average increment by 2100 (depending on the model considered) predicted by the Intergovernmental Panel on Climate Change (IPCC; http://www.ipcc.ch/) [167]. In particular, the extended Holocene period has included multiple periods of short- and long-term climate change, including a particularly steep temperature increase at the beginning of the Holocene (about 11,700 years ago) and a climate optimum reached during the mid-Holocene (Fig. 4) [168]. The Holocene also witnessed the domestication of crops in the early Neolithic of the Fertile Crescent 10,000 to 8000 years ago and in other farming centers in Asia, Africa, and the Americas.

There is much to learn from the aDNA of early crops and their propagation and local adaptation outside of their native domestication area [169]. In particular, the possibility to obtain reliable estimates of the allelic trajectory at virtually any genomic locus during the major climate transitions that have occurred in the past opens an avenue towards the identification of the genetic variants that underlie adaptation to novel environmental conditions. They could represent priority targets for breeders and/or top-candidates for reintroduction into modern germplasms.

#### Diversification

Domestication and recent breeding have reduced the genetic diversity of modern cultivated germplasm. Looking for novel sources of diversity (currently absent not only from the elite pool but also from extant wild species and landraces) is a major concern for the sustained improvement of commercial lines. aDNA can deliver the genetic diversity that has been lost at several key time points during plant domestication, with the potential of reintroducing extinct loci (gene alleles) for key traits. Mutant screening technologies offer the opportunity to validate such variants functionally prior to their reintroduction. If these variants still segregate amongst wild relatives, they could be reintroduced through conventional marker-assisted breeding/selection programs; otherwise, they could be introduced through genome editing. Ultimately, aDNA investigations would allow us to uncover the content of the ‘lost’ diversity to be resurrected in modern germplasm (a process referenced to as ‘de-extinction’), which might be of utmost value for current breeding and conservation initiatives.

## Novel scientific insights in paleogenomics that merge synchronic and allochronic approaches

**Fig. 5:**
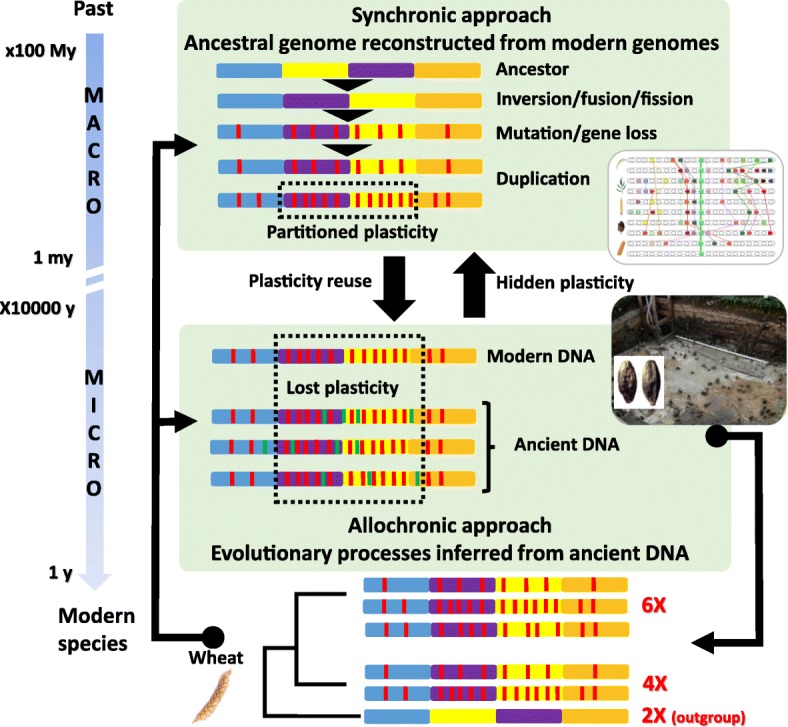
Paleogenomics from ancestral genome reconstruction and aDNA recovery. Paleogenomics encompasses a synchronic approach (*top*), involving the reconstruction of ancestral genomes of several million years old (macro-evolution) from comparisons of modern genome sequences, and an allochronic approach (*bottom*), involving the recovery and analysis of ancient DNA from archaeobotanical remains of several hundreds or thousands of years old (micro-evolution). Both approaches are complementary in unveiling the impact of past evolutionary processes (million-years-old genomic rearrangements such as duplications and inversions, illustrated with *colored blocks*) on the diversity of the modern germplasm (thousand-years-old mutations illustrated with *red* and *green vertical bars*, the latter representing mutations that have been lost during domestication and/or the adaptation of modern species). Comparison of DNA from modern and ancient diploid (2X), tetraploid (4X), and hexaploid (6X) species offers the opportunity to investigate the genomic drivers (duplication, inversion, deletion, fusion, fission, mutation…) of plant evolution and adaptation to environmental constraints, as exemplified by wheat (*bottom*). Excavation and photo A.-M. et P. Pétrequin (CRAVA, CNRS) from Clairvaux-les-Lacs (Jura), CL VII, IVth Millennium BC

The evolution of modern species can be investigated in detail through the analysis of ancient genomes. The indirect (synchronic) approach, derived from the computational reconstruction of ancestral genomes of several million years old (macro-evolution), is built on the comparison of the genomes of modern species. The direct (allochronic) approach derives from the recovery of ancient DNA from remains that are up to several tens of thousands of years old (micro-evolution). Embracing both synchronic and allochronic approaches into the growing field of paleogenomics is paramount to understanding how past macro- and micro-evolutionary processes shaped modern plant diversity (Fig. 5). Future advances can be expected in addressing whether recurrent genomic rearrangements (including polyploidization and diploidization events) have affected recent adaptation to environmental constraints and how the partitioning of genomic plasticity following polyploidization (producing stable and plastic genomic compartments) may have influenced the selective response to novel natural and/or anthropogenic pressures. In particular, the evolutionary frameworks presented above holds the potential to unveil the extent to which post-polyploidy subgenome plasticity (comparing sensitive (MF or S) and dominant (LF or D) genomic compartments) can be considered as a driving evolutionary force that provides a reservoir of novel mutations to be selected during selection or domestication in particular genomic regions. Such research questions pertaining to the role of polyploidy and partitioned genomic plasticity in the adaptive response to selection or domestication and climate change are highly novel and may provide the basis for technological innovations aimed at further developing the breeding capacity of the crop industry.

## Abbreviations

AAK: Ancestral angiosperm karyotype
aDNA: Ancient DNA
AEK: Ancestral eudicot karyotype
AGK: Ancestral grass karyotype
AMK: Ancestral monocot karyotype
CAR: Contiguous ancestral region
CCF: Centromeric chromosome fusion
GO: Gene Ontology
K–Pg boundary: Cretaceous–Paleogene boundary
mya: Million years ago
oPGs: Ordered protogenes
PPD: Post-polyploidization diploidization
pPGs: Putative protogenes
SB: Synteny block
SNP: Single nucleotide polymorphism
TCF: Telomeric chromosome fusion
WGD: Whole-genome duplication
